# Volcanic ash deposition as a selection mechanism towards woodiness

**DOI:** 10.1038/s44185-023-00018-2

**Published:** 2023-07-01

**Authors:** Carl Beierkuhnlein, Manuel Nogales, Richard Field, Ole R. Vetaas, Anna Walentowitz, Frank Weiser, Reinhold Stahlmann, María Guerrero-Campos, Anke Jentsch, Félix M. Medina, Alessandro Chiarucci

**Affiliations:** 1 Biogeography, University of Bayreuth, Universitätsstr. 30, 95440 Bayreuth, Germany; 2 Instituto de Productos Naturales y Agrobiología (IPNA-CSIC), La Laguna, Tenerife, Canary Islands Spain; 3 School of Geography, University of Nottingham, NG7 2RD Nottingham, UK; 4 Department of Geography, University of Bergen, Bergen, Norway; 5 Área de Medio Ambiente, Gestión y Planeamiento Territorial y Ambiental (GesPlan S. A.), Tenerife, Canary Islands Spain; 6 Disturbance Ecology, University of Bayreuth, Universitätsstr. 30, 95440 Bayreuth, Germany; 7 Consejería de Medio Ambiente, Cabildo Insular de La Palma, Santa Cruz de La Palma, Canary Islands Spain; 8 BIOME Lab, Department of Biological, Geological & Environmental Sciences, Alma Mater Studiorum—University of Bologna, Bologna, Italy

**Keywords:** Ecology, Ecology

## Abstract

The high proportion of woody plant species on oceanic islands has hitherto been explained mainly by gradual adaptation to climatic conditions. Here, we present a novel hypothesis that such woodiness is adaptative to volcanic ash (tephra) deposition. Oceanic islands are subject to frequent eruptions with substantial and widespread ash deposition on evolutionary time scales. We postulate that this selects for woodiness through an increased ability to avoid burial of plant organs by ash, and to re-emerge above the new land surface. We sense-checked using observations of plant occurrences and distributions on La Palma (Canary Islands) in April 2022, 4 months after the end of the eruptions of the Tajogaite volcano (Cumbre Vieja ridge). In contrast to herbs and grasses, most woody plants persisted and were already in full flower in areas with 10+ cm ash deposition. Remarkably, these persisting woody plants were almost exclusively endemics.

## Introduction

Islands contribute substantially to global biodiversity because many endemic species are restricted to individual islands or archipelagos^1^. In addition, because of their comparable origin through volcanism, discrete boundaries, and isolation from vast mainland areas, oceanic islands can be seen as model ‘natural experimental laboratories’ for understanding drivers of biodiversity^2–4^.

A series of fundamental theories, concepts and discussions have been presented recently on the evolution and morphology of island plants (e.g^5–8^.). One major question is why island floras contain such numerous woody plant species, many of which belong to families and genera dominated by herbaceous species on the mainland^9^. In several genera, woodiness evolved repeatedly^10,11^. The tendency to ‘secondary insular woodiness’ may indicate island-specific selective filtering favouring the evolution of woodiness.

Darwin^12^ was puzzled by this question, attributing island arborescence to the struggle for light. Climatic conditions such as drought have been recently promoted as drivers of woodiness^9,13,14^. However, additional biotic and abiotic drivers of trait selection may be relevant, which are not represented in global climatic data sets.

The Canarian archipelago has been studied for centuries, and excellent data are available regarding taxonomy, species distribution and endemism (e.g. refs. ^15–22^). Additionally, these islands are well monitored in terms of geology and climate, which facilitates excellent analyses of drivers influencing plant morphology and functioning. This makes the Canary Islands an excellent arena to challenge and develop theories in biogeography and evolution.

The 2021 Tajogaite volcanic eruptions on La Palma offered a unique opportunity to investigate facets of island biogeographical and even more ecological and evolutionary theories that had previously been postulated, and to develop new theories. We draw on a common feature of many islands with high endemism—volcanism—to postulate a new hypothesis to offer an additional explanation for the selection of woodiness, be it ancestral, derived, or insular. Specifically, we hypothesise that the hitherto neglected, but frequent, phenomenon of ash deposition is an important evolutionary force promoting woody growth forms on volcanic islands.

### Island rules and phenomena

Endemism is characteristic of oceanic islands and can be explained by their spatial and functional isolation^23,24^. Currently, the drivers of this island phenomenon are understood based on phylogenetic and climatic data^5,9,25,26^. The insular climate is generally rather moderate compared with continental terrestrial habitats, providing long-term stability of environmental conditions. Furthermore, the oceanic matrix reduces the difference between the maximum and minimum temperatures due to the high energy-storing capacity of water, which also influences the moisture conditions. In contrast, ocean currents, predominant wind regimes and topography cause considerable variation in local climates within many oceanic islands.

Ecologically distinctive lineages on islands are known to exhibit or lack specific morphological and behavioural syndromes or dispersal traits^27–29^. The characteristic dominance or absence of specific traits or syndromes is often explained by the lack of biotic interactions and drivers of speciation, as islands are species-poor compared with continental landscapes of the same size. Consequently, there may be a large portion of empty Grinellian niche space—so certain habitats are not occupied, mutualistic relationships such as pollination may be reduced to very few pollinators, seed dispersal systems of fleshy-fruited plants may involve different animal taxa, and antagonistic relationships such as predation or herbivory may be missing.

Paradoxically, apparent opposite effects can emerge from such deficiencies in biotic interactions between and within species. These phenomena are best understood for animals. Gigantism (disproportionally large organisms compared to related species in mainland areas) can arise from a lack of predation. Nanism (also known as dwarfism) can arise from resource limitations and the advantages of early reproduction^29^. Additionally, abiotic drivers specific to islands affect the life cycles and morphology of biota. Amongst plants, the loss of armature or other defensive traits against herbivory is common in the absence of large herbivores^30^. An obvious syndrome is the tendency towards woodiness on islands^31–35^. Woodiness (and also growth form) has been conceptually approached from ecological and evolutionary perspectives, including links to biodiversity patterns at different scales, ranging from islands to biomes^8,36,37^.

The evolution of convergent, and thus non-random, traits and growth forms is a classic and still unresolved key topic in ecology and biogeography. Insular woodiness is a common syndrome. It is found across different families and on many islands all over the globe that have been isolated during evolutionarily relevant time scales^32–34,37,38^. The Canary Islands provide examples of the evolution of secondary woodiness in many taxa independently, showcased by studies for the plant genera *Echium*^31^, *Sonchus*^39^, *Aeonium*^40^, *Sideritis*^41^, *Pericallis*^42^, *Cheirolophus*^43^ and *Euphorbia*^44^. All these taxa show substantial radiations, some considered “explosive”, within the Canarian archipelago. As a result, many endemic species have emerged, of which high proportions are endemic to single islands. Today, the genus *Echium* involves 68 accepted species (plus 4 accepted hybrids) (Plants of the World Online, accessed 17.01.2023, see databases). From 28 *Echium* species of the Canary Islands, 26 are endemics, and 25 of them are woody, despite being descended from herbaceous ancestors.

### Geological processes

Oceanic islands are a specific type of island, typically being volcanic and on, or at the edge of, oceanic tectonic plates. This contrasts with continental islands such as those located on the shelves, or continental fragments. Several main properties differentiate these two types of islands. Continental islands are of very different sizes, up to almost continental scale (e.g., Madagascar), can be very old and thus host biota that evolved over long periods of time. Continental islands are often highly diverse in parent material due to their accumulation of different rock types through Earth’s history. Oceanic islands, conversely, are relatively small, young, and formed almost completely of volcanic bedrock^45,46^. Thus, they are relatively uniform in petrography and soils, which supports global comparability and facilitates the detection of fundamental ecological processes. Both types of islands can host high mountains or mountain ranges, but on oceanic islands these are solely created by volcanic activity. These specific characteristics and processes contribute to the importance of oceanic islands as study systems for biogeography, ecology, and evolution.

Hitherto, ongoing impacts of geological processes on island biotas have been widely ignored. This contrasts with geomorphological processes that shape oceanic islands’ surfaces (e.g. refs. ^47–49^)—drivers of long-term structuring of topography are increasingly understood, such as the role of climatic conditions for erosive processes or the legacy of oceanic islands in Earth history, documented in guyots and seamounts^50–52^. However, very few studies focus on the direct influence of volcanism on the vegetation of oceanic islands^53,54^. Given that volcanic islands that developed on the oceanic crust have always been isolated from other terrestrial habitats, the parent material for soil formation stems from volcanic processes. Pedogenesis may, however, additionally be influenced by aerosols such as the dust transported from continental deserts. Nevertheless, it is volcanic eruptions that shape oceanic islands, as recently observed in the Canary Islands and in Tonga. When this active phase is terminated, erosion and landslides take over and modify the islands’ topography.

Volcanic activity has shaped the current topography of oceanic islands through its regime of repeated disturbance. Such islands are generally not created during one single phase of eruptions but exhibit ongoing volcanism over long periods of time. In the case of La Palma, the oldest volcanic rocks are dated to 1.8 million years^55^. Volcanic and other processes, including giant landslides, are well studied on this island^56–58^. Modern eruptions are documented, including in the recent past^59^, with three events taking place in the last 75 years: in 1949 (San Juan), 1971 (Teneguía) and 2021 (eruption of Tajogaite, Cumbre Vieja).

Strombolian-type eruptions are prominent volcanic processes ejecting large amounts of cinders, lapilli, ashes, and lava bombs. The repeated deposition of substantial amounts of volcanic ash is reflected in the geological structure of volcanic islands, which often show long-term alternation of tephra (ash) layers with layers of basalt. Evidently, ash deposits are a frequent phenomenon at evolutionarily relevant time scales; considering this eminent contribution to the development and dynamics of terrestrial habitats, the role of volcanic ash has not received sufficient attention^60^. Most research on pyroclastic ashes is related to atmospheric processes (e.g. Eyjafjallajökull eruption^61^,) and to large-scale impacts in Earth history^62^. In contrast to lava flows, this parent material has been widely ignored in ecological studies, for instance on primary succession^63^. Very few studies have investigated the vegetation on volcanic tephra substrate^64,65^.

**Fig. 1:**
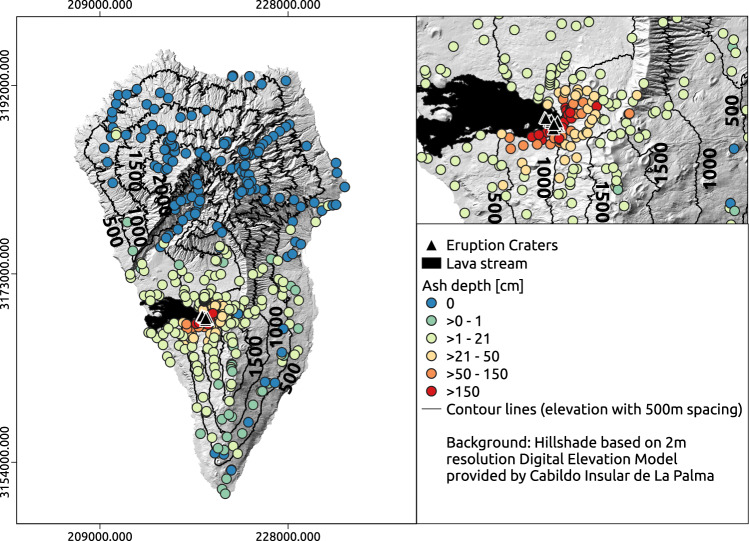
Volcanic ashes. Ash (tephra) deposition during the recent eruption of the Tajogaite volcano in 2021 on La Palma, including the depth of the newly formed ash layer, the location of the new craters, and the lava flow (own records for ash thickness).

**Fig. 2:**
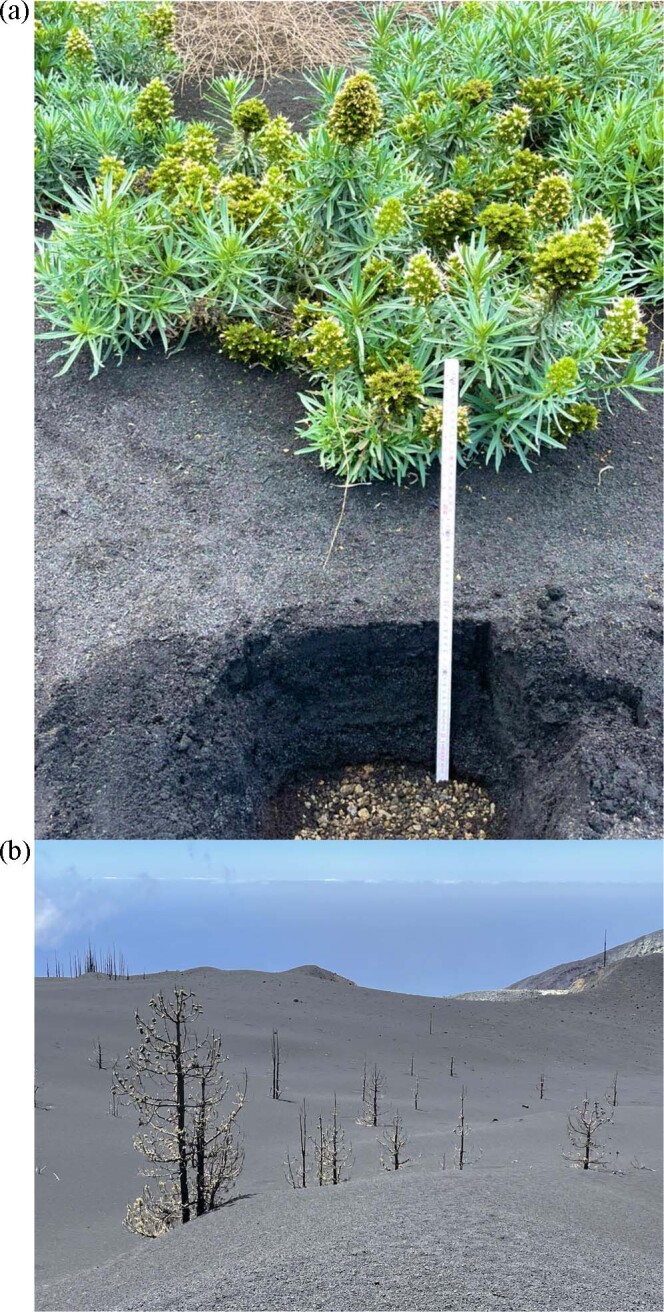
Impact and recovery. **a** Flowering Single Island Endemic shrub *Echium brevirame* at a site with 30 cm fresh ash layer, 4 months after the end of the eruptions. **b** Resprouting canopy branches of *Pinus canariensis* that were exposed to toxic sulphur gases during the eruptions, sticking out of metres-deep ash deposits. (Fotos by C. Beierkuhnlein).

A good example of the impact of these processes is La Palma, in the Canary Islands. Eruptions during the last 500 years are well documented on La Palma through historical reports^66,67^. The geological map of the island indicates basaltic and pyroclastic outcrops throughout the entire development during the Quaternary period^68^. Subrecent and undated tephra layers on the entire island indicate frequent ash deposition events, typically with considerable ash depth. The recent volcanic fissure eruption of “Tajogaite” (Cumbre Vieja) on La Palma, between 19 September and 13 December 2021, was very well monitored, including the development of volcanic cones, lava flows, and explosive activity^69–72^, and, most notably, also impacts on ecosystems^73,74^. Ash deposition was a massive phenomenon during this eruptive phase (Fig. 1). Extensive deposits of volcanic ashes covered previously developed soils and vegetation (Fig. 2). Ayris and Demelle^75^ demonstrated that precipitation can impede the erosion of deposited but not yet consolidated ash by wetting and cementation, which explains the stable surfaces of lapilli fields. Even if tephra is not a solid rock, it can predominate as a stable surface for centuries.

### Volcanic ash deposition as a selective force towards woodiness

Plants with woody stems are less physically affected by ash deposition than herbaceous species that have soft tissues and relatively low growth height. Consequently, populations of herbaceous species may tend to be more prone to extinction at local or even island scale, than populations of woody species^73^. Plants belonging to taxa with predominantly herbaceous growth forms but with capacity or pre-adaptation to woodiness are more likely to survive single events of ash deposition than more obligately herbaceous taxa. This creates positive selective pressure towards the secondary insular woodiness trait, which is likely to become predominant in the long run when ash deposition is frequent (on evolutionary timescales).

The development of woodiness in floras is considered an outstanding trait within angiosperms^14,76^. There are two quantitative foci of global plant species, in terms of functional traits, one representing herbaceous and one representing woody species^77^, indicating fundamental drivers in the evolution of angiosperms differentiating between herbaceous and woody species. The functional responses of plant traits to climate differ substantially beyond woodiness^78^. Island biota, however, only provide small proportions of global assessments and databases. Additionally, their habitats are characterized by a high degree of climatic constancy due to their small area of terrestrial habitat within an oceanic matrix. Thus, we can question which climatic drivers, beyond constancy, would explain the dominance of woodiness on islands.

Considering how frequent volcanic eruptions are in the histories of oceanic islands, ash deposition impact must be regarded as a potential major driver of selection towards traits that enable organisms to cope with, survive, or even benefit from ash deposits. In contrast to the destructive impacts (on standing biomass) caused by lava flows, ash deposits have characteristics more typical of a selective filter. A random drift of body sizes, as suggested by Biddick & Burns^6^, is unlikely to occur in the presence of such selective processes.

Although volcanic eruptions may seem infrequent relative to human perceptions, they are in fact frequent on evolutionary time scales and can re-occur within the life-cycles of long-lived perennial plants. Cursory evidence suggests that individual trees, such as the individual “patriarch” *Juniperus cedrus* tree in the Teide National Park on Tenerife, may be >1200 years, perhaps even 1500 years^79^. Such plants will have survived impacts of repeated volcanic activity. In the case of the “patriarch” tree, reported historical eruptions during its existence were reflected in the radial increment of its trunk, but clearly it survived those episodes^80^.

Different effects are expected, depending on the gradient of ash thickness. On La Palma, the 2021 Tajogaite eruptions led to volcanic ash deposition almost everywhere on the island, ranging from a thin layer that is hardly noticeable in parts of the island distant from the eruption craters to a depth of around 20 metres next to the craters^71^. Where ash deposits are thickest, almost no vegetation remained, except for tips of trees sticking out of the newly created ash surface. At intermediate distances from the craters, with ash thickness ranging between 0.25 and 1 m, no herbaceous understory vegetation emerged from this ash layer, while endemic shrub species did emerge, in profusion. Many of these shrub species exhibit secondary (insular) woodiness. However, the process of ash deposition also favours species that exhibit continental, ancestral woodiness (or pre-adaptations to it). Altogether, we found a broad spectrum of endemic woody species emerging from the ash. Most of them were vital, resprouting, and flowering <4 months after the end of the volcanic activity. These single-island endemic and multi-island endemic shrubs included species of ancestral and of derived secondary insular woodiness.

**Fig. 3:**
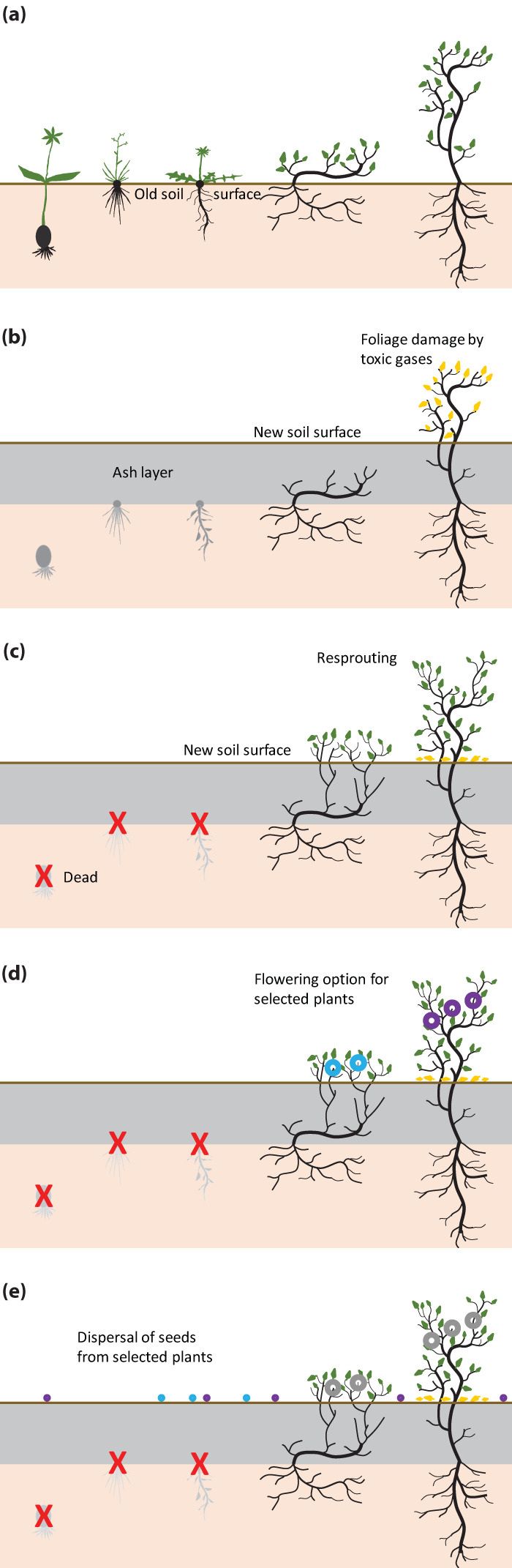
Theoretical concept. Hypothesised selection of woody plants through ash deposition. **a** Understorey plant life-form spectrum before ash deposition on developed oxidized soil. **b** New volcanic ash deposition covers previous vegetation structure (here ~0.5 m depth), causing photosynthetically active organs to die. Exposed leaves may become chlorotic (yellowish) from toxic gas impact and are likely to be shed. **c** Death of plants that cannot reach the new surface (geophytes, graminoids, forbs). These may become locally extinct (red X). Resprouting of woody plants that protrude from the ashes (trees, larger shrubs) or reach the new surface with new shoots (small shrubs). **d** flowering and reproduction of remaining species populations. **e** dispersal of propagules, germination, and establishment on the newly formed soil surface while parent plants remain alive.

Ashes can completely cover the former soil and understorey vegetation after pyroclastic eruptions. If there are no seed sources on safe sites in the vicinity, no rapid plant establishment on top of the new ash layer can be expected where the seed bank in the former topsoil is too deeply buried. Most herbaceous species cannot make it through thick layers of deposits. Therefore, we hypothesise that ash deposits function as a selective filter and evolutionary driver favouring woody species (Fig. 3) and thus also promoting the selection of the secondary (insular) woodiness trait. This process, which affects whole populations and is frequent in evolutionary time on volcanic islands, selects for perennial woody plants whose structures are still functional after ash deposition. Repeated events will increasingly support such populations.

Our ash deposition filter concept bears a striking link to the classical life form concept of Raunkiaer^81,82^ for perennial plants. Central to his concept is the position of the resprouting organs (buds, bulbs, rhizomes, seeds) during an unfavourable period (e.g., frost during winter), with respect to the soil surface, and as a second criterion to the height of the snow layer. Geophytes (with rhizomes or bulbs) endure the unfavourable period under the soil surface, hemicryptophytes (e.g., herbaceous rosette plants) right at the surface, buds of woody chamaephytes (dwarf shrubs) are covered by snow and thus are protected from harmful deep frost events, and finally, nanophanerophytes (shrubs, understorey trees) and phanerophytes (trees) are exposed to the climate all year round. This classification is still widely used even under climatic conditions where lasting snow layers during winter do not currently exist, and even short phases of deep snow (>30 cm), that would cover (and protect) chamaephytes completely, have become very rare.

The frequency and seasonal regularity of winters are fundamentally different from the kind of unpredictable impact related to volcanic eruptions, but at the time scale of long-lived woody species’ life cycles, such pulses are highly probable. Propagules of plants that show traits enabling the survival of such pulses have comparative advantages^83^. Experimental and modelling evidence suggests that ecosystem recovery after pulse disturbances is likely to be significant but incomplete, and highly dependent on the type of impact and ecosystem^84,85^.

However, most research on disturbance impacts focuses on human perturbations or on extant ecosystems of large spatial and / or economic importance, such as wildfires in boreal forests^86^. Rarer and more spatially limited impacts of natural volcanic eruptions in continental landscapes, such as the Mt. Saint Helens event, are seen as singularities as they affect only a small portion of biomes, ecosystems, and species range sizes. On islands, catastrophic volcanic eruptions that may sterilize entire islands, like the Krakatau eruption of 1883, have been considered with respect to species extinctions, new colonization, and succession^87^. On Stromboli, constant eruptive activity and ash deposition affect the island flora, which is species-poor relative to other islands of the Aeolian archipelago^88^. And even if geological processes are increasingly acknowledged to be important in shaping oceanic islands’ topography and stimulating evolution and island biodiversity through habitat diversity^48,49^, the general dynamic model of oceanic island biogeography (GDM)^47^ is focused on the general life cycle of oceanic islands but does not highlight the selective contribution of short-term impacts on speciation.

### Woodiness and endemism

The relative dominance of woodiness in island floras requires explanation. The secondary (insular) woodiness is of particulate interest because it has emerged in a broad spectrum of plant clades. This phenomenon is highly polyphyletic and homoplastic^89^. Functional and morphological patterns that are irrespective of taxonomy or phylogenetic lineages are caused by selective mechanisms. Concerning the global importance of woodiness and its legacy in Earth’s history, more than one single mechanism is likely to be the cause of it. Woodiness comes with a series of very different advantages and trade-offs.

On islands, ecological opportunity^90^ and favourable climatic conditions^24^, such as the absence of frost or the constant supply of moisture, are seen as essential drivers of morphological adaptation in island clades. However, geological processes typical to oceanic islands have been “neglected to a surprising extent” in research on secondary woodiness^91^. In addition to the constancy of long-term climatic conditions, short-term disruptive disturbance events are an evident and very characteristic feature of volcanic islands. Thus, ecological constraints through repeated disturbances can hardly be ignored. Interpreting plant traits just based on the available geoinformation background, such as extensive data on climate or island area, can be misleading. For volcanic islands on the oceanic crust, high degrees of spatial isolation and climatic constancy coincide with intermittent volcanic activity.

Repeated mechanical perturbations and a related unstable environment are mostly linked with herbaceous species characterized by high dispersal capacity and many small seeds. Species with short life cycles would also have a higher likelihood of speciation. It is expected that more “extreme” site conditions would favour short-lived herbaceous species^91^. This is inconsistent with the outstanding dominance of native and endemic woody species including ancestral woody and stem-succulent plants (Table 1)^22^. Besides dispersal, García-Verdugo et al.^92^ argue that population persistence should be considered to understand island diversification better.

**Table 1:** Number and proportion of woody and endemic plant species for the entire archipelago and the island of La Palma^22^.

	Canary Islands		La Palma	
All plant species	2417		1161	
Life-Form = Phan./Nanophan./Cham. (% of all plant species)	804	33.3%	308	26.5%
Endemic Species (% of all plant species)	608	25.2%	208	17.9%
Single Island Endemics (SIE) (% of all plant species)	359	14.9%	47	4.0%
Multi Island Endemics (MIE) (% of all plant species)	249	10.3%	161	13.9%
Endemic Woody Species (% of End. Spec.)	410	67.4%	134	64.4%
Single Island Woody Endemics (SIWE) (% of End. SIE)	269	74.9%	38	80.9%
Multi Island Woody Endemics (MIWE) (% of End. MIE)	141	56.6%	96	59.6%

The rows from ‘Life-Form’ to ‘MIE’ include percentages of all plants (first row). The last three rows include percentages of the equivalent rows above.

Here, we suggest an additional mechanism selecting for woodiness on volcanic islands, which takes into account that the frequent deposition of volcanic ashes changes the soil surface, which has until now been treated as a constant site condition. This soil surface change affects established plants dependent on their growth height and mechanical stability. The gradient of ash thickness from several metres close to volcanic cones to millimetres in remote parts of islands allows the survival, also, of herbaceous species, but repeatedly favours the selection of woodiness across large areas relative to island sizes. With ash covering a vast area around a focal plant, the advantage of extensive seed production in short-lived herbaceous species, mainly attributed to disturbed sites, does not come into effect as their seeds are simply buried and are locally taken out of the ecological and evolutionary game.

The hypothesized selective contribution of volcanic ash deposition should not be seen as the only, or even the main, driver of woodiness on volcanic islands. However, it is likely to be an additional and hitherto neglected mechanism, which favours all species with woody structures, independent of their phylogenetic origin. Evidently, multiple factors can be causes of the dominance of woody species in different ecosystems. Volcanic (oceanic) islands are a special case. However, such islands are very similar in geo-ecological processes, soil formation, nutrient availability, climate, and disturbance regimes. Their common origin on the oceanic crust and their abundance and permanent existence (as a type) during Earth’s history, requires a better understanding of selective mechanisms resulting in speciation and endemism.

## Discussion

Due to the clear spatial limitation and isolation of islands, they are classic arenas for developing ecological and evolutionary theories^42^. Different types of islands (e.g. continental, oceanic, old, young, large, small) exhibit different processes and outcomes on, for instance, plant morphology^93^. In addition to the size of geographic space and its isolation, other physical aspects such as topography and climate have been applied to explain insular endemism and woodiness with large macroecological data sets^94^. However, short-term but frequent (on evolutionary times scales) singularities such as moderate volcanic eruptions have hitherto been widely ignored. This may be because diffuse processes cannot readily be traced a posteriori in ecology, evolution, and succession research^73^.

The recent volcanic eruption on the island of La Palma yielded a large lava flow and modified the landscape of the southwestern slopes of the island. However, ash deposition affected a much larger area. Hitherto, research on the ecological impacts of pyroclastic ashes has been restricted to case studies related to continental volcanos^65,95–99^. Consequently, the contribution of such processes to the selection of plant life forms, and to endemicity, has not been considered until now. Here, we postulate that such ash deposition—a common trait of strombolian volcanic eruptions—selects for woody perennials and thus can be an (additional) explanation of the insular woodiness syndrome on oceanic islands. This mechanism is novel to the literature and to the ongoing debate about the island woodiness syndrome.

## Data Availability

Data that are referred to in this study are documented in the supplement of the FloCan checklist^22^. In addition, following databases were used: Copernicus Emergency Management Service EMSR546 (accessed on 22 June 2022)^100–103^. Atlantis Biota database (https://www.biodiversidadcanarias.es/biota/). Plants of the World Online (Kew) (http://www.plantsoftheworldonline.org/). https://volcano.si.edu/volcano.cfm?vn=383010.
